# “Proteinjury”: a universal pathological mechanism mediated by cerebrospinal fluid in neurodegeneration and trauma

**DOI:** 10.3389/fcell.2025.1593122

**Published:** 2025-05-20

**Authors:** Vladimir F. Lazarev, Bashar A. Alhasan, Irina V. Guzhova, Boris A. Margulis

**Affiliations:** Laboratory of Cell Protection Mechanisms, Institute of Cytology, Russian Academy of Sciences, St. Petersburg, Russia

**Keywords:** neurodegeneration, protein aggregates, stress, cerebrospinal fluid, toxicity, proteinjury

## Abstract

Cerebrospinal fluid (CSF) is a vital body fluid that supports the normal physiological functions of the brain and spinal cord. However, pathological conditions associated with injuries and neurodegenerative diseases lead to the accumulation of peptides, proteins, and their oligomers or aggregated forms in the CSF. In such cases, the CSF serves as a carrier and distributor of these pathogenic structures, facilitating secondary damage through the cytotoxic effects of protein aggregates. To describe this phenomenon, we introduce the term “proteinjury.” To date, accumulating experimental evidence has identified key protein complexes that contribute to proteinjury, particularly in the context of neurodegenerative diseases, traumatic brain injuries, ischemic strokes and others commonly associated with cell death and the appearance of formerly cytoplasmic proteins in the extracellular milieu. This review explores the mechanisms underlying the formation of pathogenic protein complexes in CSF, the diagnostic potential of CSF protein biomarkers, and the prospects for rehabilitation therapies aimed at preventing secondary damage mediated by pathogenic protein structures in CSF. Based on the findings discussed in this review, we conclude that proteinjury represents a universal and critical mechanism in the progression of various neurodegenerative disorders, and a deeper understanding of this phenomenon may provide new insights for the development of targeted interventions to improve clinical outcomes.

## 1 Introduction

Human biological fluids, including blood plasma, joint fluids, cerebrospinal fluid (CSF) represent a source of factors that regulate the physiological functions of the entire organism. Consequently, their analysis serves as a cornerstone of modern medical practice. Research on the composition and function of CSF came to the forefront when it was recognized as a key factor influencing the progression of various brain pathologies, ranging from post-traumatic syndrome to severe hereditary diseases (Agah et al., 2018; Dreger et al., 2022; Tsitsopoulos and Marklund, 2013; Zheng et al., 2024). During the pathogenesis of neurodegenerative diseases, such as Alzheimer’s disease (AD) (Pilotto et al., 2023; Shim et al., 2022), Parkinson’s disease (PD) (Raghunathan et al., 2022; Saijo et al., 2020), amyotrophic lateral sclerosis (ALS) (Ding et al., 2015), as well as traumatic brain injury (TBI) and strokes, factors causing so-called secondary damage accumulate in the intercellular space (Duan et al., 2024; Li et al., 2023). These factors include reactive oxygen species, (excitatory neurotransmitters (e.g., glutamate and aspartate), proinflammatory cytokines, and cytotoxic protein oligomers and aggregates (fibrils) (Lazarev et al., 2021; Yoo et al., 2022).

In this review, we introduce proteinjury, a novel pathological mechanism defined by the extracellular accumulation and cerebrospinal fluid-mediated dissemination of cytotoxic protein aggregates that perpetuate prolonged secondary neurodegeneration. Distinct from classical proteotoxic stress, which arises from intracellular protein misfolding and organelle dysfunction (Kurtishi et al., 2019), proteinjury originates from the cytotoxic activity of extracellular protein complexes, such as β-amyloid, tau, and α-synuclein oligomers, within the CSF and interstitial fluid. These aggregates arise from primary neurodegenerative processes (e.g., AD, PD), acute injuries (e.g., TBI, stroke), or aging-related clearance failure (e.g., glymphatic/blood–brain barrier (BBB) dysfunction) (Brunelli et al., 2023; Dodd et al., 2022; Mravinacová et al., 2024; Sweeney et al., 2018). Once liberated into the CSF through neuronal death or impaired clearance mechanisms, they exploit the CSF’s dynamic circulation to distribute toxicity across neural tissues, circumventing the localized cell-to-cell transmission of prion-like propagation (Frost and Diamond, 2010). Critically, the CSF’s limited capacity to clear these aggregates allows proteinjury to persist, which corroborates a spectrum of pathogenic processes, including oxidative stress, synaptic dysfunction, BBB compromise, and neuroinflammation, thereby driving prolonged secondary neurodegenerative damage throughout the central nervous system (CNS).

The chronicity of proteinjury underscores its clinical significance: pathogenic protein complexes in CSF endure long after the initial insult, fueling self-perpetuating cycles of neuronal loss and functional decline (Gaetani et al., 2020; Simon and Iliff, 2016). This phenomenon positions proteinjury as both a biomarker and a therapeutic target. By reframing secondary neurodegeneration through the lens of extracellular proteotoxicity, this concept bridges acute injury and chronic disease, providing a unified framework for understanding diverse neurological disorders.

CSF analysis has long been pivotal in diagnosing neurodegenerative diseases, with total protein levels and specific aggregates serving as sensitive indicators of pathology. Advances in proteomic and imaging technologies now enable precise characterization of CSF protein dynamics, revealing how alterations in amyloidogenic proteins, neuroinflammatory mediators, and BBB integrity correlate with disease progression. These insights not only validate proteinjury as a mechanistic entity but also highlight its translational potential for therapies targeting extracellular proteostasis in CSF.

## 2 CSF in neurodegeneration

Secondary damage to brain cells, mediated through CSF, follows a distinct trajectory, and over the past 30–40 years, CSF has served as a source of biomarkers for a wide variety of brain pathologies, such as AD, PD, ALS, TBI, and strokes (Agnello et al., 2024; Gaur et al., 2023; Heckler and Venkataraman, 2022). In addition to aggregating proteins, a large number of polypeptides are present in the extracellular space, which, in one way or another, regulate the activities of neurons, glia and neuroepithelial cells. Such polypeptides influence disease progression and serve as therapeutic targets, making them valuable biomarkers in CSF proteome research. Recent advancements in CSF analysis suggest that this biological fluid can be utilized to model a wide range of pathological conditions in cells of neuronal origin. For example, models of Alzheimer’s pathology and traumatic brain injury have been developed, and, accordingly, conduct large-scale neuropharmacological studies (Duits et al., 2024; Lazarev et al., 2021; Rykunova et al., 2023).

CSF is the only biological medium whose examination in clinical practice provides information about the state of the CNS. Many diseases of the brain and spinal cord are accompanied by alterations in the normal composition of CSF (Wright et al., 2012). One of the key components of CSF whose concentration varies in diseases of the CNS is total protein. Measuring total protein concentration in CSF is a fundamental diagnostic analysis, which is performed when infectious–inflammatory, oncological, autoimmune, or a number of neurodegenerative diseases are suspected (Felgenhauer, 1974; Merril et al., 1981). As a result, it serves as one of the most sensitive indicators of CNS pathology.

Normally, adult CSF contains 150–500 mg/L of protein (Schilde et al., 2018). Approximately 80% of CSF proteins originates from the blood filtration through the choroid plexus (CP) of the brain’s ventricles, while the remaining portion is produced intrathecally by choroid plexus cells, glia, neurons, and immune cells. The CSF protein composition is largely dependent on the protein composition of the blood. Therefore, similar to blood, albumin is the most abundant protein in CSF, comprising 35%–80% of total CSF protein. The predominant immunoglobulins in CSF belong to the class G immunoglobulins (IgG); however, it should be noted that, normally, the majority of CSF IgG originates from the blood rather than being synthesized intrathecally (Johanson et al., 2011). Despite these similarities, the CSF protein composition is markedly different from that of blood (Pezzini et al., 2023). This distinction is primarily due to the presence of the BBB, which restricts the passage of blood proteins into CSF. Small proteins like albumin easily pass through the BBB, whereas the diffusion of larger proteins, including immunoglobulins, is significantly limited. For example, albumin diffusion from blood to CSF takes approximately 1 day, whereas IgM immunoglobulin diffusion requires several days (Matsueda et al., 2018). As a result, total CSF protein constitutes only 0.1%–0.2% of total blood protein. In addition, CNS-specific proteins can be detected in the cerebrospinal fluid, such as myelin basic protein, glial fibrillary acidic protein and tau protein (Jankovska et al., 2022; Povala et al., 2021; Wang et al., 2022). These specific proteins, however, account for about 1%–2% of the total protein present in cerebrospinal fluid.

In addition to its primary role as an effective tool for analyzing and predicting the course of neurodegenerative diseases, CSF play a crucial role in the response of brain cells to pathogenic factors, therapeutic interventions, and recovery mechanisms following injury or disease. In our opinion, a special role in these processes belongs to toxic protein complexes that accumulate in CSF and can have a decisive effect on the progression of neurodegeneration. In the following sections, we summarize the existing evidence on these toxic protein complexes and their implications for both the pathogenesis and therapy of neurodegenerative disorders. Furthermore, given the distinct role and function of these toxic structures, we introduce the “proteinjury” term to characterize their pathological properties, with the aim to establish proteinjury as one of the key physiological processes mediated through CSF that contributes to nervous tissue degradation across a wide range of pathological conditions.

## 3 The concept of secondary damage

Secondary damage is a complex pathological process that follows primary injury in neurodegenerative diseases, TBI, and stroke. This condition is induced by factors that gradually accumulate in the intercellular space of the brain, including reactive oxygen species, excitatory neurotransmitters (e.g., glutamate and aspartate), proinflammatory cytokines, and cytotoxic protein oligomers and aggregates (fibrils), all of which contribute to neuronal dysfunction and disease progression (Bjorklund et al., 2023; Everse and Coates, 2009; Hiskens et al., 2023; Strogulski et al., 2023).

**FIGURE 1:**
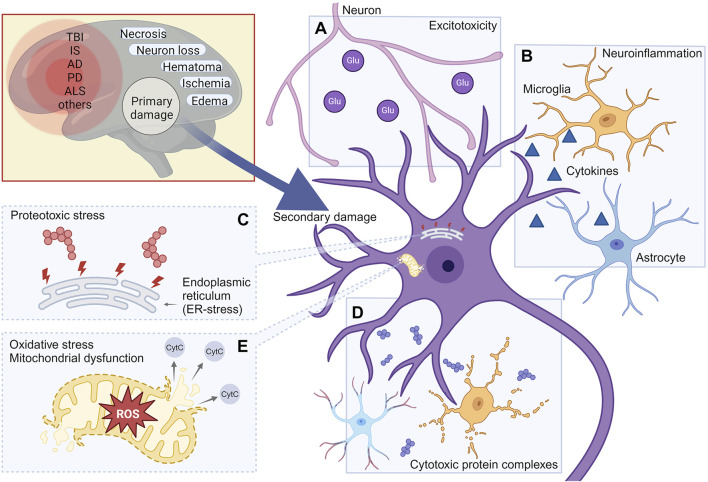
Molecular mechanisms leading to the development of secondary damage in various neurodegenerative conditions. The mechanisms of secondary damage shown in the figure include: Excitotoxicity **(A)**; Neuroinflammation **(B)**; Proteotoxic stress **(C)**; Cytotoxic protein complexes **(D)**; Oxidative stress **(E)**. TBI, Traumatic brain injury; IS, Ischemic stroke; AD, Alzheimer’s Disease; PD, Parkinson’s disease; ALS, Amyotrophic lateral sclerosis. Created with BioRender.com.

Unlike primary damage that occurs immediately following an injury, such as a stroke or TBI, and exerts a direct physiological impact within one to 2 days, secondary damage can persist for extended periods, significantly affecting cellular function and survival. Under certain conditions, secondary damage may develop over months or even several years (Arnold et al., 2022; Donaldson et al., 2023; Munoz-Ballester et al., 2022). Many studies have investigated the pathological processes underlying secondary damage, emphasizing its prolonged effects on neuronal survival and neurodegeneration. The existence of a therapeutic window during this process provides an opportunity for neuroprotective interventions aimed at preserving viable neurons. To assess the role of cytotoxic protein complexes in comparison to other contributing factors (excitotoxicity, reactive oxygen species, neuroinflammation and others), we briefly review the primary molecular mechanisms involved in secondary damage and subsequent neurodegeneration across TBI, stroke, and various neurodegenerative diseases (Figure 1).

### 3.1 Inducers of secondary damage: excitotoxicity and disruption of calcium homeostasis

Excitotoxicity refers to the process by which neuronal damage or death occurs due to the excessive activation of neurotransmitters, particularly glutamate (Figure 1A). This phenomenon arises when nerve cells are overstimulated, causing an excessive buildup of calcium (Ca^2+^) and subsequent activation of mitochondrial Cytochrome C release, ultimately triggering apoptosis. Excitotoxicity is associated with various diseases of the nervous system, including Alzheimer’s disease, Parkinson’s disease, various types of trauma and ischemic stroke (Lau and Tymianski, 2010).

CSF plays a critical role in the mechanisms of excitotoxicity, serving as a conduit for neurotransmitters and other signaling molecules between neurons. In several neurodegenerative diseases (e.g., Alzheimer’s and Parkinson’s disease), as well as acute brain injuries (e.g., ischemic stroke and trauma), studies have demonstrated that the level of glutamate in the CSF can be significantly increased (Chiang et al., 2006; Ellis et al., 2015; Olby et al., 1999), leading to excitotoxicity and neuronal damage.

Moreover, recent clinical studies investigating CSF enzymes levels involved in glutamate metabolism, such as glutamate–oxaloacetate transaminase and glutamate–pyruvate transaminase, have revealed a marked increase in their concentrations in patients following intracranial hemorrhage, leading to decreased functional activity of the brain and other neurological disorders. Furthermore, elevated levels of these enzymes were correlated with poor neurological outcomes (Snider et al., 2023). Therefore, the control of glutamate levels in CSF is essential for the treatment of neurodegenerative pathologies and traumatic brain injuries.

### 3.2 Inducers of secondary damage: neuroinflammation

Neuroinflammation represents another major contributor to secondary damage in the CNS (Figure 1B). Under normal conditions, neurons, except in specific regions like the pituitary gland, are located in an immune-privileged environment, maintained by the functions of the BBB. However, in neurodegenerative pathologies, the BBB integrity is compromised, allowing the infiltration of proinflammatory cytokines into brain tissue (Yuan et al., 2023). BBB disruption is hypothesized to be a rapid process that may initiate inflammatory cascades, involving cell adhesion, neutrophil migration, lipid metabolism and angiogenesis (Bowman et al., 2018). This phenomenon is a hallmark of various neurodegenerative diseases and post-traumatic conditions, such as after TBI or ischemic stroke (Aragón-González et al., 2022).

A common stage involved in all designated pathological conditions is the activation of microglia, accompanied by the infiltration of leukocytes from the periphery through the damaged BBB, transepithelial migration, and diapedesis (Hinson et al., 2015; Lee et al., 2014). The inflammatory responses following TBI and ischemic stroke is largely mediated by the release of proinflammatory cytokines, such as tumor necrosis factor (TNF), interleukin-1 (IL-1) and interleukin-6 (IL-6), as well as the upregulation of cell adhesion molecules, such as Intercellular adhesion molecule 1 (ICAM-1) (Hang et al., 2005).

The evaluation of CSF and brain tissue from TBI patients and rodent models have demonstrated the presence of polynuclear leukocytes and cytokines like interleukin-1β, interleukin-6, and TNFα within 24 h after injury. In addition, the damaged parenchymal endothelium exhibits increased synthesis of adhesins, such as ICAM-1 and vascular cell adhesion molecule-1 (VCAM-1), during this period, which recruit leukocytes from the periphery to the injury site (Worthylake and Burridge, 2001). The infiltrating leukocytes further disrupt the integrity of the BBB through the generation of ROS, activation of proteolytic enzymes, and secretion of cytokines and chemokines (Chodobski et al., 2011; Winkler et al., 2016).

Depending on the balance of pro- and anti-inflammatory signals, the immune response can either promote recovery from injury or cause further damage to nerve tissue (Jickling et al., 2015). Thus, despite the fact that proinflammatory cytokines are not pathogenic proteins in the canonical sense, their appearance in brain tissue provokes the death of neurons and contributes to disease progression (Fraga et al., 2019).

### 3.3 Inducers of secondary damage: proteotoxic stress and cytotoxic protein structures

Proteotoxic stress, caused by proteins with disrupted conformations, including oligomers and aggregates, is a critical factor that significantly impact long-lived neurons. Such stress can be induced by both endogenous (Figure 1C) and exogenous proteins and their complexes (Figure 1D).

Many neurodegenerative diseases are clearly associated with the aggregation of specific proteins. For instance, it is well established that in Alzheimer’s disease, aggregates (senile plaques) are primarily composed of tau protein and β-amyloid peptide. In Parkinson’s disease, Lewy bodies are formed based on α-synuclein (Sung and Chung, 2025), while in Huntington’s disease, aggregates are formed by the mutant huntingtin protein (Cho, 2025). Research into these diseases has further demonstrated that protein aggregates may contain other proteins, including those essential for the normal functioning of nerve cells (Akkentli et al., 2024; Upadhyay A. et al., 2023). Notably, protein aggregation is not restricted to classical neurodegenerative disorders; several studies have shown that protein aggregates also form during non-specific neurodegenerative processes, such as those occurring after TBI or ischemic stroke (Dutysheva et al., 2022; Lazarev et al., 2018; Wu et al., 2022).

A common feature across these pathological conditions is that protein oligomers and aggregates are found both intracellularly and in the intercellular space. In addition, various *in vivo* and vitro models have demonstrated that these protein complexes have, to a greater or lesser extent, cytotoxic effects on neuronal cells (Dutysheva et al., 2020; Lazarev et al., 2021; Rykunova et al., 2023). Among aggregation-prone proteins, tau is one of the most frequently implicated in the formation of toxic complexes. Thus, tauopathies, which are characterized by the formation of tau deposits, have been observed in TBI (Alyenbaawi et al., 2021), ischemic stroke (Hayden et al., 2019), Alzheimer’s disease (Congdon et al., 2022), Parkinson’s disease (Meloni et al., 2023), Huntington’s disease (Baskota et al., 2019), ALS (Montalbano et al., 2020) and many other disorders.

Another well-known protein prone to forming aggregates is α-synuclein, whose oligomerization and aggregation are traditionally associated with the progression of Parkinson’s disease, specifically with the dysfunction of synapses (Murphy and McKernan, 2022). However, recent emerging evidence has convincingly demonstrated the tendency of α-synuclein to participate in the formation of aggregates in Alzheimer’s disease (Jin et al., 2022; Shim et al., 2022), ALS (Koch et al., 2016), and following TBI (Rokad et al., 2017; Sauerbeck et al., 2021) and ischemic stroke (Rokad et al., 2017).

In addition to well-characterized pathogenic aggregation-prone proteins such as α-synuclein, tau, and β-amyloid, conditionally normal proteins can also modulate the toxicity of protein aggregates in neurodegenerative processes. One such protein is glyceraldehyde-3-phosphate dehydrogenase (GAPDH), a key enzyme in the glycolytic cycle. However, GAPDH has been shown to significantly enhance the cytotoxic effect of aggregates formed by mutant huntingtin (Lazarev et al., 2015; Mikhaylova et al., 2016) and β-amyloid (Lazarev et al., 2021), and it is itself capable of forming toxic aggregates under oxidative stress (Lazarev et al., 2016) and also following TBI (Lazarev et al., 2018; Lazarev et al., 2020).

It is important to note that not all protein aggregates are inherently toxic; and their impact on neurodegeneration is nuanced by factors such as aggregate conformation, size, and biological context. For example, Benilova et al. demonstrated that highly purified infectious prions retain full seeding activity but are not directly neurotoxic to cultured neurons. Moreover, they showed that neurotoxicity in infected brain homogenates could be eliminated without reducing infectivity, supporting the idea that infectivity and toxicity are mechanistically distinct processes (Benilova et al., 2020). This finding has important implications for understanding extracellular aggregation in neurodegeneration, and underscores that toxicity is not solely determined by the ability of aggregates to propagate, but by their specific structural and biochemical properties.

### 3.4 Inducers of secondary damage: reactive oxygen species

Reactive oxygen species (ROS) are highly reactive molecules that contribute to the pathology of secondary TBIs through oxidative stress mechanisms (Figure 1E) (Rodriguez-Rodriguez et al., 2014). One such mechanism involves the activation of Ca^2+^-dependent nitric oxide (NO) synthetases and phospholipases, leading to the production of reactive nitrogen and oxygen forms (XIONG et al., 1997). During normal metabolism, superoxide anions (O_2_^−^) are generated and subsequently converted into hydrogen peroxide (H_2_O_2_). In turn, H_2_O_2_ serves as a precursor for hydroxyl radicals, which are among the most reactive chemical groups. When a hydroxyl radical combines with a nitrite ion, they form a peroxynitrite, a potent oxidant. These reactive oxygen and nitrogen species induce widespread damage by triggering pathological oxidative modifications in lipids, proteins, DNA, membrane structures and mitochondria (Massaad and Klann, 2011).

Elevated ROS levels in CSF can lead to oxidative stress and damage the cells, including neurons. This process contributes to the development of inflammatory processes, the activation of apoptosis (programmed cell death) and the degeneration of nerve cells, all of which are implicated in the progression of neurodegenerative diseases and deterioration of patient’s conditions (Gibson et al., 2008; Ruan et al., 2016). At the molecular level, lipid peroxidation, nuclear and mitochondrial DNA damage, and increased levels of protein oxidation markers have been detected in both the brain and CSF of patients with Alzheimer’s disease at various disease stages (Mecocci et al., 1997; Schippling et al., 2000). In this case, ROS can be transported through the CSF to different parts of the brain, spreading oxidative damage and exacerbating secondary damage.

Thus, CSF can accumulate a variety of cytotoxic factors, primarily protein-based, throughout an individual’s lifetime. Collectively, these factors exert a significant influence on neurodegenerative processes. Focusing on the protein component of secondary damage, the following section explores the mechanisms regulating proteostasis in CSF.

## 4 Systems for maintaining protein homeostasis in the intercellular space of the brain

Throughout the normal function of neurons, various protein molecules and metabolic byproducts appear in the intercellular space. Meanwhile, protein complexes circulating in the blood and lymph are typically restricted from penetrating brain tissue. However, during neurodegenerative processes, protein oligomers and aggregates have been shown to appear in the interstitial space. The utilization and removal of these cellular waste products is carried out using protein clearance systems, which must ensure their transport into the CSF and subsequently into the blood and lymphatic circulation. These clearance system become dysfunctional under pathological conditions, leading to the accumulation of protein aggregates in both brain tissue and CSF. The accumulation and persistence of these protein structures within the intercellular space induces neuronal death.

**FIGURE 2:**
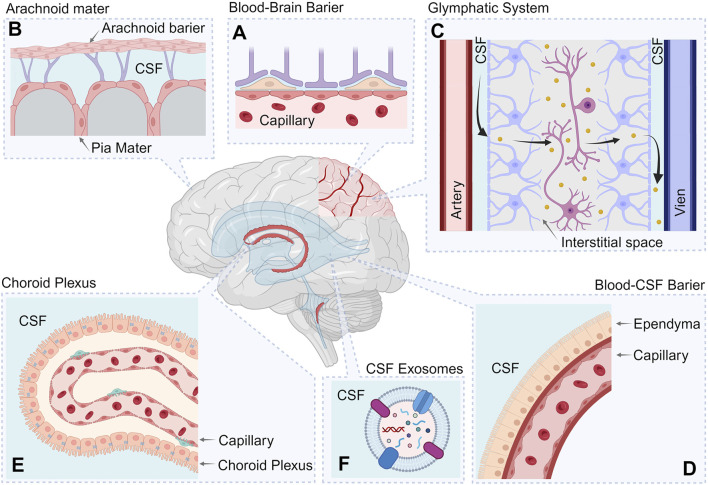
Systems involved in maintaining protein homeostasis in CSF. The figure shows the following processes involved in protein clearance: Blood–brain barrier **(A)**; Arachnoid and Pia maters **(B)**; Glymphatic System **(C)**; Blood-Cerebrospinal fluid barrier **(D)**; Choroid Plexus **(E)**; CSF exosomes **(F)**. Created with BioRender.com.

At the same time, in addition to the removal of biomolecules from brain tissue into the CSF and systemic circulation, a counterflow mechanism exists, allowing certain molecules to travel from the blood into CSF and subsequently into brain tissue. This bidirectional process is regulated by the same barrier systems, including the BBB and blood–CSF barrier (BCSFB) (Xue et al., 2023) (Figure 2).

The following sections consider the key components of the protein clearance system and their functioning features in various neurodegenerative conditions. Focusing on the protein component of secondary damage, the following section explores the mechanisms regulating proteostasis in CSF.

### 4.1 Blood–brain barrier

The blood–brain barrier (BBB) is a critical component in maintaining the specialized protein environment required for neuronal function (Figure 2A). Its primary function is to protect the brain from toxins, microorganisms and humoral protein factors circulating in the blood, while also ensuring the effective clearance of waste products from nervous tissue into the bloodstream. In some contexts, the term “BBB” is used to refer to multiple barriers simultaneously, including the blood–brain barrier itself, the blood–CSF barrier, the choroid plexus, and the barrier formed by the arachnoid membrane. However, in this review, these structures will be considered separately.

In neurodegenerative pathologies or following injuries and strokes, the normal function of the BBB may be disrupted (Canepa and Fossati, 2021). For example, it has been shown that experimental TBI can lead to the destruction of the BBB in mice, which leads to a partial loss of the immune privilege of nervous tissues (Chen W. et al., 2023), and notably, such disorders can persist for extended periods. In patients, immunohistochemical staining for fibrinogen and immunoglobulin G (IgG) has revealed persistent BBB damage more than a year post-injury (Hay et al., 2015).

At the same time, long-term BBB damage has also been associated with increased risk of developing dementia (Abrahamson and Ikonomovic, 2020). Moreover, clinical studies investigating brain capillary damage and soluble platelet-derived growth factor receptor-β (PDGFR-β) suggest that increased BBB permeability to blood-derived proteins may serve as an early biomarker of cognitive dysfunction in humans (Moon et al., 2023; Nation et al., 2019). Preclinical studies have further demonstrated that the long-term disruption of BBB permeability led to increased accumulation of β-amyloid deposits in mice neurons. Disruptions in BBB permeability should be thus considered a key driver of long-term secondary damage, including that mediated by pathogenic proteins (Acharya et al., 2024).

The opposite is also true. As a rule, the development of neurodegenerative processes can lead to the BBB disruption. For example, in Parkinson’s disease, the appearance of α-synuclein aggregates in the culture medium of hCMEC/D3 human brain endothelial cells has been demonstrated to reduce these endothelial cells viability (Hourfar et al., 2023). In a mouse model of ALS and frontotemporal dementia, loss of the TAR DNA-binding protein 43 (TDP-43) in endothelial cells resulted in BBB dysfunction, including increased barrier permeability (Cheemala et al., 2023).

Likewise, β-amyloid has been shown to contribute to increased BBB permeability on immortalized human cerebral microvascular endothelial cells (Parodi-Rullán et al., 2020). A similar phenomenon was observed in Tg-SwDI mice (Rosas-Hernandez et al., 2020), where the authors linked the increased BBB permeability with the influence of β-amyloid deposits on the enhanced expression of the receptor for advanced glycation end products (RAGE) protein in BBB cells.

It is evident that BBB damage accompanying neurodegenerative conditions of diverse origins will inevitably lead to alterations in CSF composition (Tumani et al., 2018). These alterations include both qualitative and quantitative changes in CSF protein composition. Importantly, BBB dysfunction exacerbates the development of secondary damage, including that mediated by the action of pathogenic proteins and their complexes. Simultaneously, the accumulation of cytotoxic protein complexes in CSF can further disrupt BBB function, establishing a vicious cycle of neurodegeneration.

### 4.2 Arachnoid and pia maters

The role of the arachnoid and pia mater in maintaining the protein composition in the CSF (Figure 2B) includes the following aspects (Adeeb et al., 2013; Ghannam and Kharazi, 2023; Go, 1997):

– Barrier function: The pia mater serves as an important biological barrier between the brain’s neural tissues and CSF, regulating and controlling the passage of various molecules and substances, including proteins, between CSF and the tissues around the nervous system.
– Filtration and metabolism: The arachnoid membrane acts as a passive filter that selectively allows certain molecules to pass through its cells and facilitating substance exchange between CSF and adjacent tissues, including proteins.
– Regulation of protein composition: The arachnoid membrane may participate in regulating the levels of proteins and other molecules in CSF, helping to maintain protein homeostasis of the fluid that is essential to ensure the physiological functioning of the CNS.

While the role of these meninges in the development of neurodegeneration has not yet been sufficiently studied, their damage in acute injuries, such as TBI, has been well documented. Moreover, TBI-induced meningeal injury has been shown to cause the accumulation of angiopoietin proteins, which in turn regulate the permeability of the BBB (Chittiboina et al., 2013).

### 4.3 Glymphatic system

Since it discovery in 2012 by M. Nedergaard and colleagues, the glymphatic system has been recognized as a key component involved in the clearance of waste products from the CNS tissues into the interstitial space (Iliff et al., 2012; Yang et al., 2013). This system ensures the flow and filtration of fluids, CSF and water, through brain tissues, directing them from the periarterial space through the interstitial space into the perivenous space (Figure 2C). Thus, the glymphatic system ensures the interchange of components between the interstitial fluid and CSF (Lohela et al., 2022).

It is believed that the protein aquaporin 4 (AQP4) plays a key role in the glymphatic system functioning, forming water-conducting channels on the membrane of astrocytes (Naganawa and Taoka, 2022; Simon et al., 2022). Suppression of AQP4 expression in mice has been shown to disrupt the entire glymphatic system function, primarily due to impaired interstitial fluid flow (Gomolka et al., 2023).

Notably, as with many protective physiological systems, the glymphatic system degrades with age, resulting in slower clearance of the interstitial space and the accumulation of insoluble protein aggregates, which can also provoke the development of dementia (Kress et al., 2014; Nedergaard and Goldman, 2020). Given that the glymphatic system is largely associated with CSF, nearly all neurodegenerative processes are likely to contribute to the accumulation of protein aggregates in both the interstitial space and CSF.

### 4.4 Blood–CSF barrier

The barrier between blood and CSF, the blood–cerebrospinal fluid barrier (BCSFB), is a complex system that, along with the BBB, protects the CNS from potentially harmful substances present in the bloodstream (Figure 2D) (Saunders et al., 2023; Zhang et al., 2022). In general, the functions and purpose of the BCSFB are essentially similar to those of the BBB; however, a key distinction lies in the sheer number of transporters that exchange water, solutes and peptides between the blood, neural tissues, and CSF.

Endothelial transporters primarily transport glucose, amino acids, and free fatty acids across the BBB to nearby neurons and glia (Johanson, 2017). In contrast, BCSFB transporters supply plasma glucose and organic acids for local epithelial CP metabolism, while also delivering growth factors, hormones and peptides into the CSF, ensuring their global distribution throughout the brain (Huttunen et al., 2022; Wang and Zuo, 2018). The BCSFB consists of specialized cells that form the structural elements of blood vessels in the brain and spinal cord. These endothelial cells are characterized by specific proteins and compounds that control the passage of substances from the blood into the CSF and *vice versa* (Spector et al., 2015; Tumani et al., 2018).

It is important to note that in neurodegenerative conditions, the most common consequence of BCSFB dysfunction is increased permeability and the abnormal transport of specific inflammatory proteins (Seeliger et al., 2024). Notably, the elevated expression and transport of proinflammatory factors in BCSFB correlate with increased disease severity, as demonstrated in studies involving patients with moderate cognitive impairment and advanced dementia (Ott et al., 2018). In a mouse model, it was hypothesized that β-amyloid oligomers may disrupt the integrity of the BCSFB by activating matrix metalloproteinases (MMPs) in CP epithelial cells (Brkic et al., 2015).

Similarly, after acute injuries such as TBI or hyperthermia, both BCSFB damage and increased permeability are observed, leading to increased brain swelling. Interestingly, the use of neurotrophic factors such as Cerebrolysin, brain-derived neurotrophic factor (BDNF), and glial cell line-derived neurotrophic factor (GDNF) has been shown to alleviate the increased permeability of BCSFB, and, accordingly, reduce brain edema and restore sensorimotor deficits in injured rats (Sharma et al., 2010; Sharma and Johanson, 2007).

### 4.5 Choroid plexus

Another critical structure essentially involved in maintaining protein homeostasis in CSF is the choroid plexus. The choroid plexus consists of a network of capillaries located within the ventricles of the brain, where it produces CSF by filtering blood and releasing fluid directly into the brain’s ventricles (Saunders et al., 2023). This is achieved by specific cells, such as choroidal epithelial cells, which form a selective barrier between the bloodstream and CSF (Figure 2E). This barrier allows for the selection of molecules that pass from the blood to the CSF and *vice versa* (Hutton et al., 2022).

The choroid plexus plays a crucial role in filtering the blood and regulating the production and composition of CSF (Huff and Varacallo, 2019; Johanson et al., 2011). It is involved in the transport of water, electrolytes, glucose and other molecules, as well as in the exchange of proteins between the blood and CSF. Normally, immunoglobulins, transport proteins, cytokines and growth factors enter the CSF through the choroid plexus. Given this function, it is not surprising that diverse neurodegenerative disorders in the brain adversely impacts the function of the choroid plexus, leading to dysregulation of CSF biomolecular composition, primarily affecting its protein content (Chen Y. et al., 2023).

Thus, the maintenance of proper protein homeostasis in CSF relies on multiple systemic mechanisms, including barrier systems, clearance pathways, and protein expression regulation. However, under pathological conditions, the function of these systems becomes compromised, leading to the accumulation of toxic proteins and their complexes in CSF. In the following sections, we explore the mechanisms underlying the accumulation of pathogenic protein structures in CSF.

## 5 Accumulation of proteins in CSF in pathological conditions

During neurodegenerative processes due to aging, injuries or strokes, a wide range of completely different protein structures can accumulate in CSF. These include native proteins, as well as denatured, pathogenic proteins, oligomers, and aggregates. However, the mechanisms underlying the appearance of such aggregates in CSF and their subsequent disposal remain incompletely understood. This section examines the key factors contributing to the accumulation of protein structures in CSF.

### 5.1 Proteins accumulation in CSF during neurodegenerative processes

In neurodegenerative diseases such as Alzheimer’s disease and Parkinson’s disease, aggregated proteins are frequently detected in CSF, which include aggregates composed of β-amyloid, α-synuclein, and TDP-43, among others (Audrain et al., 2023; Gan et al., 2023; Lazarev et al., 2021). The origins of these protein aggregates in CSF may vary, with the most probable cause being their release from the intercellular space following neuronal cell death, with their subsequent movement from the interstitial space into CSF contributes to their accumulation. Additionally, barrier dysfunction at the interface between CSF and blood can severely disrupt proteostasis, further intensifying the accumulation of pathogenic proteins and aggregates in CSF.

Dysregulated protein clearance mechanisms, when coupled with barrier disruption, may facilitate the formation of amyloid plaques, neurofibrillary tangles, or other cytotoxic protein structures, all of which induce neuronal damage and cell death (Giunta et al., 2007; Lazarev et al., 2021; Smith et al., 2024). This process is often accompanied by inflammation and the activation of immune system.

The accumulation of denatured or pathogenic proteins and their complexes in CSF can also disrupt normal metabolism and neurotransmitters in brain, which, in turn, further accelerates the progression of neurodegenerative processes and deterioration of the patient’s condition (Di Maio et al., 2023; Zuo et al., 2017). Thus, CSF protein analysis, such as measuring biomarkers associated with neurodegenerative diseases, may serve as an essential tool for diagnosis and disease monitoring.

### 5.2 Accumulation of proteins in CSF following ischemic stroke

After an ischemic stroke, the cerebral blood circulation is disrupted, leading to nerve cells death and tissue damage, triggering inflammatory response and immune system activation, which contribute to the accumulation of proteins in CSF (Daniel et al., 2025; Thom et al., 2016).

As part of the post-stroke inflammatory cascade, various cytokines, chemokines, and other inflammatory mediators are released and may infiltrate CSF due to blood–brain barrier (BBB) dysfunction (Li et al., 2017; Naik et al., 2023). Additionally, proteins associated with axonal injury and neuronal damage may accumulate in CSF, with some of these proteins being also capable to form aggregates. Notably, in certain cases, the accumulation of specific proteins, particularly ferritin, has been implicated in exacerbating oxidative stress and neurotoxicity (Gámez et al., 2021). Therefore, analyzing CSF protein composition following ischemic stroke may offer valuable insights into its pathophysiology, aids in diagnosis and prognosis, and may contribute to the development of therapeutic and rehabilitation strategies for affected patients.

### 5.3 Accumulation of proteins in the CSF due to TBI

Similar to previous neurodegenerative conditions, TBI induces a range of biochemical and pathological changes in CSF, including protein accumulation. This occurs due to several physiological processes triggered in response to injury:

1. Inflammation: TBI is frequently accompanied by a robust inflammatory response, leading to the release of various cytokines, chemokines and other inflammatory mediators. These molecules can cross the damaged BBB and accumulate in CSF (Clausen et al., 2019; Ma et al., 2020; Rafie et al., 2024).
2. Bleeding: TBI can cause intracranial or subarachnoid hemorrhage, introducing blood-derived proteins into CSF following BBB disruption (Csuka et al., 1999; Yan et al., 2014).
3. Necrosis and apoptosis: Neuronal and glial cell death post-injury, occurring via necrosis or apoptosis, results in the release of intracellular proteins that accumulate in CSF (Jiang et al., 2020; Santacruz et al., 2021).

These processes can contribute to the formation of protein aggregates in CSF, including those already present in CSF. For instance, aggregated forms of tau have been found in patients after TBI (Marklund et al., 2021), along with α-synuclein (Su et al., 2010), β-amyloid 1–42 (Olsson et al., 2004) and the glycolytic enzyme GAPDH (Lazarev et al., 2018).

Consequently, studying CSF protein content following TBI may provide valuable insights into injury severity, prognosis, and potential therapeutic and rehabilitation strategies (Andersson et al., 2024; Stukas et al., 2023).

In summary, it is obvious that the accumulation of proteins in CSF, including aggregated and oligomeric forms, can arise from diverse pathological processes. While some of these processes are unique and disease-specific, the majority appear to be universal features of neurodegenerative disorders. Key contributors include BBB dysfunction and impaired protein clearance systems, both of which are characteristic of nearly all neurodegenerative conditions. As a result, proteins and protein complexes that are uncharacteristic of normal CSF physiology accumulate, leading to the formation of subsequent cytotoxic structures.

Within the framework of this discussion, it can be assumed that the formation of toxic structures based on conditionally normal proteins is due to the CSF’s lack of proteostasis systems found in neuronal cells, such as molecular chaperone, autophagy, and proteolytic systems. In the extracellular space, these functions must be carried out by CSF protein clearance systems, which often fail under pathological conditions. Given the diagnostic significance of protein alterations in CSF, deviations in protein composition and aggregation have long attracted the attention of researchers and clinicians. As a result, CSF analysis remains a critical tool for disease diagnosis and monitoring, which will be explored in the following sections.

## 6 Protein markers of diseases, including pathogenic proteins

Alterations in total protein concentration in CSF are frequently observed in conformational diseases that are caused by the accumulation of unfolded or misfolded proteins. An increase in CSF total protein levels can occur under three primary conditions: 1) Increased BBB or other barrier permeability, which is the most common mechanism underlying CSF protein changes in neurodegenerative, infectious, and inflammatory diseases, as well as in TBI and stroke (Lindblad et al., 2021; Muszyski et al., 2017; Wu et al., 2020). In such cases, CSF protein composition reflects changes in blood protein composition. 2) Intrathecal protein synthesis activation, such as in the case of multiple sclerosis, sarcoidosis or neurosyphilis (Gao et al., 2024). 3) Impaired CSF protein resorption through the arachnoid granulations, as seen in certain neurological disorders (Holman et al., 2005; Papaiconomou et al., 2002).

An elevated CSF total protein level is a hallmark of CNS pathology and is detected in various types of meningitis, neuroborreliosis, neurodegenerative diseases, TBI, and ischemic stroke. However, total protein elevation alone lacks disease specificity and cannot serve as a specific diagnostic marker (Berezovsky et al., 2017; Djukic et al., 2017).

In the following sections, we explore well- established and emerging protein biomarkers that have been linked with the progression of neurodegenerative processes and may hold diagnostic and prognostic value.

It is expected that numerous research groups are actively investigating protein markers associated with various neurological disorders, often focusing on proteins directly linked to the pathogenesis of specific diseases. A summary of well-characterized CSF protein markers associated with specific pathological processes is provided in Table 1.

**TABLE 1:** Protein markers of neurodegenerative diseases found in CSF.

Pathology	Protein marker	References
Alzheimer’s disease	β-amyloid 1–42	Jonaitis et al. (2024), Roy et al. (2024), Seidu et al. (2024)
	pTau	Jonaitis et al. (2024), Seidu et al. (2024)
	tTau	Agah et al. (2024)
	SNAP25	Halbgebauer et al. (2022), Kivisäkk et al. (2022), Nilsson et al. (2024)
	NPTX2	Nilsson et al. (2024)
	GFAP	Liu T. et al. (2023)
	Neurogranin	Kivisäkk et al. (2022)
	PSD95	Kivisäkk et al. (2022)
	Neurofilament proteins	Vrillon et al. (2024)
Parkinson’s disease	α-synuclein	Fernandes Gomes et al. (2023), Liu Y. et al. (2023), Majbour et al. (2022)
	Neuronal pentraxins	Nilsson et al. (2023)
	Neurofilament proteins	Sheng et al. (2022)
	SNAP25	Bereczki et al. (2017)
	GFAP	Liu T. et al. (2023)
	Apolipoprotein E	Upadhyay N. et al. (2023)
Prion diseases	PrP	Barria et al. (2018)
	SNAP25	Halbgebauer et al. (2022)
	t-tau	Coban et al. (2023); Senesi et al. (2023)
	14-3-3 proteins	Senesi et al. (2023)
Inflammation	IL-1β, IL-6, TNF-α, IFN-γ, IL-12p70, IL-10, IL-8	Nwachuku et al. (2016), van Amerongen et al. (2024)
TBI	Ubiquitin C-terminal hydrolase	Mondello S et al. (2012)
	S100B	Pfortmueller et al. (2016)
	GFAP	Gill et al. (2018), Shahim et al. (2020b)
	β-amyloid	Franz et al. (2003)
	Tau	Franz et al. (2003), Öst et al. (2006)
	Neurofilament proteins	Coppens et al. (2023), Martínez-Morillo et al. (2015), Shahim et al. (2020a)
	HMGB1	Au et al. (2012)

To date, many disease-associated proteins have been identified that, when dysfunctional, contribute to the development of Alzheimer’s disease (AD). These include β-amyloid, tau, and apolipoprotein E, among others. Additionally, mutations in genes such as presenilin 1 (PSEN1), presenilin 2 (PSEN2), and triggering receptor expressed on myeloid cells 2 (TREM2) are recognized as genetic risk factors. Thus, a broad panel of diagnostic biomarkers has been established for AD. Beyond β-amyloid and tau (Molinuevo et al., 2018), several neuronal inflammation markers can be also secreted during AD that demonstrate potential diagnostic value, including BDNF, insulin-like growth factor 1 (IGF-1), vascular endothelial growth factor (VEGF), transforming growth factor β 1 (TGF-β1), monocyte chemoattractant protein-1 (MCP-1), interleukin-18 (IL-18), as well as neurofilament light chain (NfL), a protein whose appearance in CSF or blood signals axonal injury. However, despite these advances, research continues to focus on the identification of novel biomarkers and the development of improved diagnostic approaches (Coppens et al., 2023).

Among recently identified AD biomarkers, AD-associated neuronal thread protein (AD7C-NTP) has gained attention. This protein co-localizes with tau neurofibrillary tangles and appears in body fluids long before the formation of neurofibrillary fibers, making it a promising early diagnostic marker (De la Monte and Wands, 2001; Jin and Wang, 2021). To detect AD7C-NTP, a “sandwich” ELISA kit was developed for urine-based testing (Ma et al., 2016). While this method was proposed as cost-effective and accurate, the study authors excluded individuals with kidney disease, raising concerns about potential false-positive results (Ma et al., 2016). Most commercially available diagnostic kits for AD are based on the “sandwich” ELISA technique, such as the “Beta-Amyloid (1–42)-ELISA EUROIMMUN” kit (Thijssen et al., 2021) and «Simoa® Aβ42 Advantage Kit-Quanterix» (Hall et al., 2022).

Scientific publications increasingly propose the use of laboratory tests capable of detecting multiple biomarkers simultaneously for diagnostic purposes, such as microchip-based assays (Park et al., 2021). One such test, the QPLEX^TM^ Alz plus assay, offers multi-target analysis for AD markers in blood, reporting an accuracy exceeding 80%. However, as the authors noted, this system required further validation on larger patient cohorts. Subsequent optimization and expanded testing improved the assay’s sensitivity to 69.4% and specificity to 90.6% (Kim et al., 2021). However, the high cost of such multi-marker approaches remains a limitation, reinforcing the ongoing research for a single, highly specific and accurate AD biomarker.

Another potential CSF biomarker for AD is the GAPDH-Aβ complex, which as previously shown, the number of its aggregates increased in patients CSF as the severity of the disease progressed (Lazarev et al., 2021). However, no current data support its use as a definitive AD diagnostic marker.

With the development of neural networks, studies have attempted to correlate protein expression patterns or levels with the prognosis of disease progression. For example, a recent study demonstrated that clustering of tau-associated proteins (NRGN, GAP43, and SNCB) or β-amyloid-associated proteins (PTPRN2, NCAN, CHL1, NCAM1, and L1CAM) significantly enhanced the predictive accuracy of AD development (Limbrick et al., 2021; Mravinacová et al., 2024). Similar prediction approaches are also being explored in the context of brain cancer, particularly in improving the early diagnosis of gliomas (He et al., 2023).

Another promising diagnostic strategy involves the use of nanopore-based sensing with molecular constructs capable of selectively binding to proteins of interest or their oligomers. This approach has already demonstrated efficacy in the diagnosis and prognosis of Parkinson’s disease, employing nanopore constructs that capture α-synuclein (Liu Y. et al., 2023). A similar technique has shown potential for β-amyloid in CSF of patients with AD using nanopore particles based on zinc oxide (Lee et al., 2019).

Modern analytical techniques have made it possible to study the profile of biomolecules in CSF as putative markers of pathologies. Among the most commonly studied biomolecules are lipids and proteins, with their relative ratio also playing a critical role in disease assessment. This parameter, as recently shown using Fourier-Transform Infrared Spectroscopy, can accurately predict the development of ALS in patients (Dučić and Koch, 2023).

As an example of the diverse methods employed to detect specific protein molecules in CSF for diagnostic purposes, the Protein Misfolding Cyclic Amplification technique is particularly noteworthy. It has been proposed that, by using this method, the presence of prion protein (PrP) in CSF of patients with human prion diseases can be determined with high accuracy, although it requires extended analysis times (up to 96 h) (Barria et al., 2018).

In addition to “classical” neurodegenerative pathologies, emerging evidence suggests that aggregates of certain proteins in CSF may indicate psychological disorders in patients. For instance, the number of DISC1 protein aggregates in CSF was significantly higher in patients with first-episode psychosis, with even greater levels in patients with schizophrenic disorders (Pils et al., 2023; Suryawanshi and Tanaka, 2023).

Another example of pathogenic protein accumulation in CSF is the appearance of cytotoxic transthyretin oligomers and aggregates in transthyretin amyloidosis (Lorenzoni et al., 2023; Takahashi et al., 2022). In this pathology, transthyretin expression is initially observed in the liver and choroid plexus, but its oligomers are found in the blood and CSF of affected individuals (Ali et al., 2023; Basanta et al., 2024).

Thus, it is now well established that, disease-specific pathogenic protein markers accumulate in CSF across a range of neurodegenerative pathologies (Ghaith et al., 2022). Importantly, monitoring CSF protein levels enables early diagnosis and tracking the progression of neurodegenerative processes, which can aid in the development of targeted therapeutic approaches and predicting the outcome of the disease (Bivona et al., 2023; Huang et al., 2024). In many cases, such protein markers serve not only as diagnostic tools but also as promising therapeutic targets due to their distinct cytotoxicity.

On the other hand, as indicated in Table 1, a major challenge in modern CSF-based protein biomarker diagnostics to date is the lack of highly disease-specific markers whose presence in CSF uniquely indicates a specific disease. Instead, many biomarkers are primarily regarded to confirm a certain pathogenic process characteristic of various neurodegenerative processes. For example, the appearance of cytokines in CSF indicates the development of an inflammatory process. While the detection of proteins such as SNAP25 and NPTX signifies axonal injury.

We believe that this “universality” of protein biomarkers in CSF arises from the fact that, regardless of initial trigger of secondary damage, exogenous protein complexes lead to neuronal and glial cells damage and the release of relatively universal set of biomarkers into CSF, which may primarily indicate cellular damage and the development of secondary damage processes.

## 7 Known mechanisms of protein-mediated CSF toxicity

One of the consequences of protein aggregate accumulation in CSF is their potential toxic effect on neuronal cells, as well as their propagation throughout brain tissue in a prion-like manner (Herman et al., 2022; Tan et al., 2015). Many studies have demonstrated CSF from patients with various neurodegenerative disorders can induce the complexation of aggregate-prone proteins *in vitro* and *in vivo* (Hirohata et al., 2011; Skachokova et al., 2019). Despite substantial evidence confirms the ability of CSF-derived protein aggregates to stimulate the pathological process of aggregation data regarding their direct toxic effect remain relatively limited. This topic was previously introduced in the section devoted to proteotoxic stress; here, we discuss this issue more comprehensively.

It is known that CSF aggregates can exert direct toxic effects on nerve cells. For example, β-amyloid oligomers and aggregates exhibit cytotoxicity on various brain structures, both within CSF and the interstitial space. β-Amyloid induces oxidative stress in neuronal cells (S. Y. Park and Chae, 2007) via the inhibition of XIAP protein (Tanaka et al., 2006) and disrupting the cell membranes of neurons (Chang et al., 2013). Additionally, β-amyloid is known to impair protein clearance structures, such as the choroid plexus (Bartolome et al., 2020) and BBB (Chan et al., 2018; Petrushanko et al., 2023), further stimulating neurodegenerative processes.

An extremely important and interesting aspect of β-amyloid cytotoxicity was raised by Zaretsky et al., who found that high levels of β-amyloid-containing complexes in CSF correlate with intensive leaching of these structures from the interstitial space (Zaretsky et al., 2022). The authors suggest that the rate of β-amyloid clearance through CSF may serve as a novel pathophysiologically significant biomarker of AD, as well as a predictor of the late onset of the disease. Similar properties have been confirmed for tau protein, another known hallmark of AD, as it has been shown that CSF-derived tau of AD patients can induce neuronal cell death *in vitro* (Jankeviciute et al., 2019).

In addition to their direct toxic effects, aggregates in CSF can indirectly affect the survival of nerve cells. Perhaps, most work has been published on the prion-like distribution of aggregates. For instance, in one of the first studies in this field, CSF from patients with frontotemporal dementia was able to induce the aggregation of the TDP-43 protein in human U251 glioma cells (Ding et al., 2015). The formation of TDP-43 aggregates led to the upregulation of cleaved caspase-3, p53, LC3II/LC3I, and Beclin-1 proteins, indicating the activation of apoptosis and autophagy. Moreover, similar aggregates could be obtained by introducing artificial TDP-43 aggregates into CSF samples from patients (Scialò et al., 2020).

Thus, even a concise review of the contemporary literature highlights that cytotoxic protein structures in CSF are a common pathological feature across various neurodegenerative disorders (Table 2). This phenomenon may be considered a universal mechanism of secondary neurodegeneration in a certain sense. Accordingly, it is necessary to take this phenomenon into account when developing novel therapeutic strategies, i.e., medications are needed to neutralize or block the toxic effects of protein structures accumulated in CSF.

**TABLE 2:** Proteins and their aggregates shown to exert cytotoxic effect when present in CSF.

Pathology	Cytotoxic protein structure	References
Alzheimer’s disease	Tau aggregates	Jankeviciute et al. (2019)
	β-Amyloid aggregates	Tanaka et al. (2006), Sancesario et al. (2018)
	GAPDH- β-Amyloid co-aggregates	Lazarev et al. (2021)
Parkinson’s disease	α-synuclein	Smith et al. (2024)
	β-Amyloid aggregates	Murakami et al. (2019)
	Tau aggregates	Murakami et al. (2019)
TBI	GAPDH aggregates	Lazarev et al. (2018)
	Tau aggregates	Marklund et al. (2021)
ALS	TDP-43 aggregates	Audrain et al. (2023)
	SOD1 aggregates	Cristóvão et al. (2013), Lehmann et al. (2020)
Frontotemporal dementia	TDP-43 aggregates	Ding et al. (2015), Scialò et al. (2020)
Amyloidosis	Transthyretin	Park et al. (2019), Sant’Anna et al. (2013)
Prion disease	Prion protein	Wei et al. (2012)

## 8 Proteinjury phenomenon as a key element of secondary damage

In the context of studying the mechanisms underlying secondary damage in neurodegeneration, it is essential to examine proteins, peptides, and their complexes or aggregates that accumulate in the extracellular space and exert toxic effects on neurons and glial cells. To capture this unique extracellular phenomenon, we have introduced the term proteinjury to describe the toxicity arising specifically from extracellular protein aggregates in the CSF. By coining “proteinjury” (rather than “protein injury”), we emphasize a mechanistically defined process characterized by persistent extracellular accumulation in CSF due to aging-related diseases, injuries, or strokes; impaired clearance mechanisms (e.g., glymphatic dysfunction); and barrier failure (BBB/BCSFB disruption) (Brunelli et al., 2023; Dodd et al., 2022; Mravinacová et al., 2024; Sweeney et al., 2018), all of which are universal, prevalent, and pathophysiologically significant in secondary neurodegeneration (Moda et al., 2023).

A key motivation for introducing proteinjury is to differentiate it from other protein aggregation mechanisms, such as intracellular proteotoxic stress, ER stress, and prion-like propagation. Intracellular proteotoxic and ER stress refer to the accumulation of misfolded proteins within cells due to failing quality-control systems (Hetz and Saxena, 2017; Kurtishi et al., 2019). In contrast, proteinjury denotes the sustained cytotoxic effects of extracellular aggregates in the CSF, which accumulate in the intercellular space and compromise neuronal/glial function through direct mechanisms (e.g., membrane disruption, oxidative stress) rather than templated misfolding. Similarly, while prion-like propagation emphasize the templated seeding and spread of misfolded proteins between cells (Frost and Diamond, 2010; Stopschinski and Diamond, 2017), proteinjury extends beyond this framework by reflecting the sustained cytotoxic effects exerted by disseminated extracellular aggregates through both the interstitial space and CSF that directly disrupt cellular functions. For instance, in Alzheimer’s disease, β-amyloid oligomers in CSF not only seed further aggregation but may also directly impair neuronal membranes and amplify inflammatory cascades (Lazarev et al., 2021; Milà-Alomà et al., 2020). Similarly, extracellular GAPDH aggregates may persist in CSF for months, driving delayed neurodegeneration through chronic exposure, creating a toxic microenvironment that induces neural degeneration, and cognitive dysfunction (Lazarev et al., 2021). Notably, proteinjury’s persistence may also stem from the CSF’s limited capacity to clear these protein aggregates (M. J. Simon and Iliff, 2016), enabling secondary damage to propagate across the CNS. In Table 3, we provide a detailed comparison to differentiate proteinjury from other protein aggregation terminologies in neurodegeneration.

**TABLE 3:** Comparative table of proteotoxic aggregation mechanisms in neurodegeneration.

Features	Intracellular proteotoxic stress	Prion-like propagation	Proteinjury (CSF-mediated extracellular toxicity)
Origin/inducer	Triggered by cellular stressors (e.g., oxidative stress, mutations) that induce misfolding within cells	Initiated by the release of misfolded proteins from damaged cells, triggering templated seeding	Results from protein aggregates release into CSF following aging diseases and injuries, or from impaired clearance mechanisms (e.g., glymphatic dysfunction, BBB/BCSFB breakdown)
Mechanism of action in neurodegeneration	Misfolded proteins accumulate in intracellular compartments (ER, cytosol, mitochondria), overwhelming chaperone, proteasome, and autophagy systems	Misfolded extracellular proteins interact with nearby cells, inducing conformational conversion and misfolding of native proteins	Toxic protein aggregates accumulate in the CSF and exert direct cytotoxic effects on neurons and glia; CSF distributes aggregates to distant CNS sites
Primary vs. secondary damage	Primary damage; early initiating event	Primarily primary damage via cell-to-cell spread	Predominantly secondary damage; prolonged, extracellular toxicity mediated via CSF
Spatial action	Intracellular; local to affected neurons or glia	Extracellular; local intercellular propagation (adjacent cells)	Extracellular; long-range toxicity mediated by CSF distribution across CNS
Overlaps and interconnections	Initial trigger that may release aggregates contributing to extracellular mechanisms (prion-like propagation and proteinjury)	Shares overlap with intracellular stress (source of aggregates); can feed into CSF pool, overlapping with proteinjury	May originate, but not exclusive, from intracellular release or seeding activity; defined by sustained toxicity via CSF, not misfolding templating
Resulting neuropathologies	Alzheimer’s disease, Parkinson’s disease, Huntington’s disease, ALS, others	Prion diseases, Alzheimer’s, Parkinson’s, frontotemporal dementia (FTD)	Secondary damage in Alzheimer’s, traumatic brain injury (TBI), ischemic stroke, neuroinflammatory states, others
Therapeutic strategies	Enhancing chaperones, autophagy inducers, proteasome activators	Blocking seeding activity, immunotherapies targeting extracellular aggregates	Neutralizing aggregates in CSF by monoclonal antibodies and small molecules, enhancing CSF clearance (e.g., glymphatic function), and restoring barrier integrity
References	Hetz and Saxena (2017), Kurtishi et al. (2019), Menzies et al. (2015), Zheng et al. (2016)	Frost and Diamond (2010), Stopschinski and Diamond (2017), Zampar et al. (2024)	Brunelli et al. (2023), Gaetani et al. (2020), Lazarev et al. (2021), Moda et al. (2023), Mravinacová et al. (2024), Simon and Iliff (2016), Sweeney et al. (2018)

**FIGURE 3:**
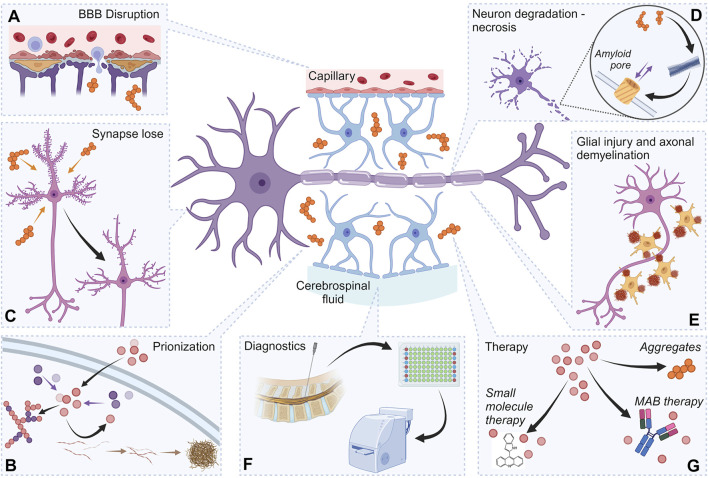
Proteinjury as a target for neurodegeneration diagnostics and therapy. The figure shows the important elements of proteinjury, as well as the role of these components in approaches to therapy and diagnostics. Destruction of synapses by protein aggregates **(A)**. Damage to the BBB involving protein aggregates **(B)**. Prion-like action of protein structures, including prionization of intracellular proteins **(C)**. Initiation of the necrotic process in neurons by exogenous protein structures **(D)**. Damage to glial cells (various mechanisms) **(E)**. Exogenous protein complexes localized in the intercellular space are used as diagnostic markers **(F)**. Exogenous protein complexes localized in the intercellular space can serve as therapeutic targets **(G)**. Created with BioRender.com.

Thus, proteinjury encompasses multiple well-characterized pathological processes, positioning it as a relevant target for both diagnosis and therapy (Figure 3), offering opportunities to mitigate secondary neurodegeneration at its extracellular origin (Gaetani et al., 2020). Below, we discuss some of the key pathological aspects involved in proteinjury in greater detail.

### 8.1 Damage and loss of synapses

One of the most important and widespread elements of neurodegenerative processes is damage and degradation of synapses, which entails functional disorders of the nervous system (Figure 3A). A major cause of synapse destruction is the presence of monomeric and oligomeric forms of β-amyloid and α-synuclein, which induce synaptic damage indirectly by activating pro-inflammatory cytokines such as TNF-α (Illes et al., 2020; Wiatrak et al., 2023; Zelek et al., 2024; Zhang and Hölscher, 2020). In addition, there is evidence of the direct effect of extracellular α-synuclein fibrils on synapse degradation, although the precise mechanisms underlying this effect remain incompletely understood.

### 8.2 BBB Disruption

BBB damage is a hallmark of neurodegenerative disorders of various origins. To date, a growing body of evidence indicates that exogenous protein aggregates contribute to BBB damage, thereby amplifying secondary neurodegeneration (Figure 3B) (Petrushanko et al., 2023; Soto-Rojas et al., 2021). This phenomenon has been well-documented for β-amyloid oligomers and fibrils, α-synuclein oligomers (Shaltiel-Karyo et al., 2013), superoxide dismutase (SOD) aggregates (Haorah et al., 2011) and other proteins prone to aggregation (Haorah et al., 2007). BBB impairment not only facilitates the entry of neurotoxic molecules into the CNS but also corroborate neuroinflammatory and degenerative processes.

### 8.3 Prion-like action

Over the past 10–15 years, a substantial evidence has accumulated indicating that most mutant proteins causing conformational neurodegenerative pathologies are capable, under certain circumstances, of exerting a prion-like effect on native intracellular proteins (Figure 3C). Specifically, these pathogenic proteins can penetrate cells, inducing the aggregation and co-aggregation of normally folded proteins (Holmes and Diamond, 2012; Ren et al., 2009). This phenomenon has been demonstrated for β-amyloid (Lacoursiere et al., 2022; McAllister et al., 2020), tau (Kfoury et al., 2012), α-synuclein (Fujita et al., 2023; Zampar et al., 2024), mutant SOD (Sproviero et al., 2018), mutant TDP-43 (Udan-Johns et al., 2014), and numerous other proteins (Mikhaylova et al., 2016).

### 8.4 Neuronal damage

The ability of mutant proteins with damaged conformation, along with their oligomeric and aggregated forms, to induce neuronal death (Figure 3D) has been well-documented previously in the literature (Arantes et al., 2025; Cordeiro et al., 2025). These cytotoxic effects occur through multiple mechanisms, including necrosis (Dutysheva et al., 2021; Lazarev et al., 2018; Lazarev et al., 2021). One of the most probable mechanisms of such toxic action involves the formation of pores in the neuronal membrane by oligomers of these proteins, which, accordingly, leads to a disruption of membrane integrity and loss of function (Gilbert et al., 2014; Matthes and de Groot, 2023; Mrdenovic et al., 2021).

### 8.5 Glia damage

Naturally, exogenous pathogenic protein complexes are capable of damaging and killing not only neurons, but also glial cells (Figure 3E). A common consequence of this process is demyelination of neuronal axons, which can lead to disruption in the transmission of nerve impulses (Traiffort et al., 2021; Yamaguchi et al., 2024). This toxic effect on glial cells has been confirmed for β-amyloid (Lloyd et al., 2021), tau (Gratuze et al., 2021), α-synuclein (Yu et al., 2018), mutant huntingtin (Jing et al., 2021), mutant TDP-43 (Smethurst et al., 2020) and several other proteins (Lin et al., 2021).

### 8.6 Diagnostics

The presence of specific protein and peptide complexes in the intercellular space, CSF, and other body environments serves as a key diagnostic marker (Figure 3F) for multiple neuropathologies (Donadio et al., 2021; Scialò et al., 2020), starting from post-mortem analysis of the patient’s brain and ending with CSF analysis after a lumbar puncture (Bargar et al., 2021; Saijo et al., 2019).

### 8.7 Therapeutic strategies

Given their significant role in disease progression, pathogenic extracellular protein structures represent promising therapeutic targets (Figure 3G) (Galasko et al., 2024). Strategies currently under investigation include the use of small-molecule inhibitors as well as therapeutic monoclonal antibodies, both of which have already undergone preclinical and clinical testing (Imbimbo et al., 2023; Lazarev et al., 2018; Lazarev et al., 2021).

Another important aspect to consider is the long-term impact of secondary damage caused by the formation and accumulation of protein complexes. Numerous studies indicate that the negative effects of protein aggregates can persist for months or even years, continuously exerting cytotoxic effects on neuronal and glial cells. For instance, protein aggregates containing β-amyloid and GAPDH from the CSF of AD patients have been shown to induce cytotoxicity in human neuroblastoma SH-SY5Y cells. Notably, these toxic effects were already evident during the moderate stage of AD and became significantly more pronounced as the disease progressed to the severe stage (Lazarev et al., 2021).

Similarly, in Parkinson’s disease, CSF from patients containing α-synuclein oligomers has previously been demonstrated to exert strong inhibitory and cytotoxic effects on cultured human astrocytes and microglia *in vitro* (Schiess et al., 2010).

Our recent work on rat CSF following severe TBI further supports this phenomenon, as GAPDH-based aggregates that formed shortly after injury and accumulated in CSF maintained their toxic effects on cultured rat C6 glioma cells for at least 2 months (Dutysheva et al., 2022).

These findings allow us to conclude that protein aggregates localized in the intercellular space, particularly in CSF, may sustain neurotoxicity on brain cells of patients for extended periods that can last for years.

In conclusion, nearly all known molecular mechanisms of secondary damage, beyond their direct induction of cell death, also lead to the emergence of proteins with disrupted conformations, including those prone to amyloid-like and amorphous oligomer formation. Thus, given its persistent and widespread impact, proteinjury may represent a master factor and possibly the longest-acting mechanism driving secondary damage in neurodegenerative processes, apparently of almost any origin.

## 9 Discussion

The concept of proteinjury introduced in this review represents a critical advancement in understanding the universal mechanisms underlying secondary neurodegeneration. By synthesizing evidence from diverse neurodegenerative diseases, TBI, and ischemic stroke, we propose that cytotoxic protein aggregates in CSF (including oligomers, fibrils or amorphous protein complexes) serve as a common pathological driver, perpetuating neuronal and glial damage long after the initial insult. This discussion elaborates on the implications of proteinjury, its interplay with established mechanisms of neurodegeneration, and its potential as a therapeutic target.

The accumulation of pathogenic protein aggregates in CSF—such as β-amyloid, tau, α-synuclein, and TDP-43—emerges as a hallmark of proteinjury. These aggregates, whether disease-specific or conditionally toxic (e.g., GAPDH), propagate neurotoxicity through multiple pathways: synaptic disruption, BBB compromise, oxidative stress, and prion-like seeding (Figure 3). Notably, proteinjury extends beyond intracellular proteotoxic stress, emphasizing the extracellular milieu’s role in neurodegeneration. For instance, CSF-derived β-amyloid and tau aggregates directly impair neuronal membranes and induce apoptosis, while α-synuclein oligomers exacerbate neuroinflammation by activating microglia and astrocytes. The persistence of these aggregates in CSF, as demonstrated in post-TBI and AD models, underscores their long-term cytotoxic potential, often spanning years.

Proteinjury intersects with well-characterized mechanisms of neurodegeneration, such as excitotoxicity, neuroinflammation, and oxidative stress. For example, BBB disruption—a common feature in AD, PD, and TBI—facilitates the bidirectional leakage of pathogenic proteins between blood and CSF, creating a vicious cycle of barrier dysfunction and aggregate accumulation (Figure 2). Similarly, the glymphatic system’s failure to clear interstitial waste, exacerbated by aging, allows toxic proteins to persist in CSF, amplifying secondary damage. This synergy suggests that proteinjury is not an isolated phenomenon but a convergent pathway that amplifies existing pathologies.

The detection of protein aggregates in CSF has revolutionized neurodegenerative disease diagnostics. Table 1 highlights CSF biomarkers like pTau and NfL, which correlate with disease progression. However, the lack of disease-specific markers remains a challenge, as many proteins (e.g., SNAP-25, GFAP) reflect generalized neuronal injury rather than distinct pathologies. Emerging technologies, such as nanopore-based sensors and multi-analyte assays (e.g., QPLEX^TM^ Alz plus), promise enhanced specificity by detecting oligomeric forms of pathogenic proteins.

Therapeutically, proteinjury presents a dual target: preventing aggregate formation and neutralizing existing toxic species. Monoclonal antibodies against β-amyloid (e.g., aducanumab) and tau exemplify this approach, though their efficacy in modifying CSF protein dynamics remains debated. Small-molecule inhibitors targeting GAPDH aggregation or enhancing chaperone activity (e.g., Hsp70 inducers) offer alternative strategies. Notably, rehabilitative therapies that restore BBB integrity or boost glymphatic clearance—such as Cerebrolysin or aquaporin-4 modulators—may mitigate proteinjury by addressing its systemic roots.

While the proteinjury framework provides a unifying perspective, several gaps persist. First, the origin of CSF aggregates—whether from neuronal death, blood leakage, or *de novo* extracellular aggregation—requires clarification. Second, the role of non-canonical proteins (e.g., transthyretin, DISC1) in proteinjury warrants exploration, particularly in non-Alzheimer’s dementias. Third, longitudinal studies are needed to establish causal links between CSF protein profiles and clinical outcomes, especially in early disease stages.

## 10 Conclusion

The accumulation of protein factors in CSF capable of initiating secondary damage represents a critical stage in various neurodegenerative processes. This pathological mechanism warrants extensive investigation and the development of novel therapeutic strategies. Unlike intracellular proteotoxic stress, which occurs within neurons, the toxic effects of secondary injury mediated by extracellular protein aggregates in CSF constitute a distinct phenomenon that we define as “proteinjury.”

Proteinjury is a universal and prolonged mechanism mediating secondary neurodegenerative damage in the brain, with the detrimental impacts potentially persisting for years or even throughout a patient’s lifetime.

The analysis of the literature presented in this review allows us to draw two major conclusions. First, a substantial evidence has been accumulated confirming the physiological significance of proteinjury, highlighting the cytotoxic effects of pathogenic protein structures in CSF on neuronal and glial cells. Second, despite its clear role in disease pathology, there are currently no specific pharmacological interventions designed to counteract proteinjury.

Considering the findings previously discussed, we conclude that therapeutic approaches targeting secondary damage induced by CSF pathogenic protein accumulation and their complexes should be broad-spectrum and applicable across neurodegenerative disorders of various origins. Developing such treatments is urgently needed and underscores proteinjury as a promising and critical target for future neuropharmacological research.
